# Developing Novel Beta-Secretase Inhibitors in a Computer Model as a Possible Treatment for Alzheimer's Disease

**DOI:** 10.1155/adpp/5528793

**Published:** 2025-03-31

**Authors:** Tassanee Ongtanasup, Komgrit Eawsakul

**Affiliations:** ^1^Department of Applied Thai Traditional Medicine, School of Medicine, Walailak University, Nakhon Si Thammarat 80160, Thailand; ^2^Center of Excellence in Tropical Pathobiology, Walailak University, Nakhon Si Thammarat 80160, Thailand; ^3^Research Excellence Center for Innovation and Health Products (RECIHP), Walailak University, Nakhon Si Thammarat 80160, Thailand

**Keywords:** ADMET, Alzheimer's disease, beta-secretase, molecular docking, molecular dynamics

## Abstract

Alzheimer's disease (AD) is a neurological condition that causes neurons and axons in the brain to deteriorate over time and in a specific pattern. The enzyme beta-secretase-1 (BACE-1) plays a crucial role in the onset and progression of AD. In silico approaches, or computer-aided drug design, have become useful tools for reducing the number of therapeutic candidates that need to be evaluated in human clinical trials. Finding chemicals that bind to BACE-1's active site and inhibit its activity is key for preventing AD. A pharmacophore model was developed in this study based on potent BACE-1 inhibitors previously identified, and subsequently employed to screen a commercially available compound database for similar compounds. ZINC35883784 was identified with high binding affinities and hydrogen bonding interactions. Moreover, similar properties to donepezil were found in a compound made by altering the structure of ZINC35883784 called (4R,5R)-2-[1-(2-ethylcyclohexyl)ethyl]-4-hydroxy-5-(4-hydroxybutyl)cyclohexanolate (M4). Compounds were tested for interactions with BACE-1 and favorable properties. Binding scores were confirmed after molecular docking. The assessment of drug-likeness was conducted utilizing Swiss ADME analysis. Molecular dynamics simulations assessed the stability of compound interactions with BACE-1. MMPBSA calculated binding free energy and contribution energy. Results showed that M4 had strong and steady interactions with BACE-1. M4 was also analyzed by predicted NMR and retrosynthesis. However, further experiments are needed to evaluate M4's potential as a BACE-1 inhibitor.

## 1. Introduction

Alzheimer's disease (AD) is a progressive and fatal brain disorder that has devastating effects on one's ability to remember, think, and act normally. One of the leading causes of dementia in the elderly is AD, for which there is now no treatment. German physician and neuropathologist Alois Alzheimer discovered the illness in 1906 [1]. AD is characterized by the buildup of tangles and plaques in the brain, which causes the death of nerve cells and other brain tissue [2]. As the disease advances, affected individuals may experience challenges in carrying out daily activities and may need assistance with basic self-care. While symptoms of AD typically manifest in one's sixties [3], they may also present themselves in one's forties or fifties [4, 5]. The timely diagnosis and treatment of AD can lead to a significant improvement in the quality of life for affected individuals.

The risk of developing AD has been linked to many genes. This gene provides instructions for making a protein called apolipoprotein E (APOE), which helps move fats across the body's circulatory system. The APOE gene's E4 allele is the strongest predictor of developing AD [6]. Those who have at least one copy of the APOE4 allele in their DNA are at a greater risk for acquiring the disease than those who have no copies. Although having the APOE4 allele increases your risk of developing AD, it is important to note that this is not a certainty [7, 8]. In addition to a family history of AD, additional proteins have been linked to the disease's progression. AD is thought to be caused in part by beta-amyloid (Aβ) and tau. From the amyloid precursor protein (APP), the enzyme beta-secretase (BACE1) generates beta-amyloid (Aβ) [9]. When it builds up in the brain, it may cause the production of amyloid plaques [10], which are thought to contribute to the death of nerve cells and subsequent deterioration in cognitive function that is typical of AD. The protein tau is crucial for the stability of microtubule-based structures in neurons [11]. Neurofibrillary tangles, which are aggregation of tau protein, are a major cause of cell death in AD [12]. AD is also characterized by the presence of these tangles. Although both beta-amyloid (Aβ) and tau proteins are believed to play a role in the pathogenesis of AD, it remains uncertain which of the two is the primary causative factor.

There is presently no treatment for AD that is universally accepted as the gold standard [13, 14]. The efficacy of a certain medication may vary based on the patient's condition and its stage. Several medications have been shown to be particularly helpful in reducing AD symptoms and improving patients' quality of life. Donepezil, rivastigmine, and galantamine are cholinesterase inhibitors that have been demonstrated to be beneficial in treating mild to moderate AD symptoms [15, 16]. These drugs operate by elevating levels of acetylcholine, a crucial neurotransmitter for memory and cognition. N-methyl-D-aspartate (NMDA) receptor antagonist, such as memantine, is also used to treat mild to severe AD [17]. It functions by adjusting glutamate neurotransmitter activity in the brain, which can minimize dementia symptoms and enhance general performance [18]. However, cholinesterase inhibitors like donepezil, rivastigmine, and galantamine are used in mild to moderate Alzheimer's treatment, whereas NMDA receptor antagonists like memantine are used in severe instances. The therapeutic benefits of these drugs are confined to the elevation of acetylcholine levels and regulation of glutamate activity, which are neurotransmitters in the brain. In contrast, BACE1 inhibitors target a distinct element of the illness process. BACE1 is responsible for the initial breakdown of the APP, which leads to the formation of the beta-amyloid (Aβ) peptide. The accumulation of this protein fragment is associated with AD and is believed to contribute to the development of the condition. It has been suggested that BACE1, which catalyzes the first stage of the APP degradation process, might be a therapeutic target for AD [19, 20]. BACE1 inhibition is hypothesized to decrease beta-amyloid (Aβ) formation and so slow or prevent AD development. There are several BACE1 inhibitors in clinical studies at the moment. Several of these inhibitors have exhibited promising outcomes in animal research; however, clinical trials are necessary to determine the safety and efficacy of these treatments for AD. While BACE1 inhibitors have shown promise as a treatment for AD, more research is needed to determine whether or not they are safe and effective. AD pathogenesis relies heavily on BACE1, an enzyme that proteolytically cleaves APP into smaller peptides like beta-amyloid (Aβ) peptide. Several drugs that inhibit BACE1 activity have been discovered in recent years. A number of BACE1 inhibitors are now being tested in human therapeutic studies; they include donepezil [21, 22], verubecestat [23], lanabecestat [24], elenbecestat [25], LY3202626 [26], GSK933776 [27], and E2609 [28].

In silico approaches are simulation and modeling techniques that are employed in the pharmaceutical development process. These techniques can give useful insights into the characteristics and behavior of prospective drug candidates, therefore accelerating and enhancing the process of drug discovery and development [29–34]. In this study, in silico approaches are employed for pharmaceutical development due to several key benefits. Firstly, Virtual screening: In silico approaches may be used to efficiently and rapidly screen a large number of prospective drug candidates, identifying those with the most promising features for further development. Additionally, these approaches can be used to anticipate the structure of possible drug candidates and their interactions with target proteins, offering information into how to create medications with the most desirable features. Furthermore, in silico methods may be used to predict the ADME and pharmacodynamics of potential drug candidates, providing important information about the potential for harm or benefit from these medicines. Lastly, these approaches can be used to anticipate the possible toxicity of potential drug candidates, aiding in the identification of those with the lowest likelihood of generating adverse side effects. Overall, in silico approaches provide a cost-effective and efficient tool for evaluating new drug candidates and gaining insights into their characteristics and behavior, therefore accelerating and enhancing the drug discovery and development process.

Therefore, the goal of this research was to use in silico strategies to create new chemicals that selectively inhibit BACE1 with high bioavailability and low toxicity.

## 2. Materials and Methods

### 2.1. Pharmacophore Modeling

This study used the CavityPlus web application (https://www.pkumdl.cn:8000/cavityplus/computation.php) [35] to locate cavities and connection points, predict allosteric sites, and assess the likelihood of covalent ligand binding. It obtained 3D protein models from the PDB database and identified the amino acid residues that made up the structural structure of the cavity. It used specific criteria in the cavity search, such as separated minimum depth, maximum abstract limit, and minimum abstract depth. The program predicted cavity score, pKd, and drug score using experimental binding affinity data. The CavPharmer application was then used to identify allosteric and possible covalent interactions and search for pharmacophore measurements. CavPharmer generated pharmacophore features such as a hydrophobic core, hydrogen bond donor and acceptor, positive and negative central points, and excluded volume, and was used to improve the more druggable cavity obtained from the cavity search. After identifying the binding site on the BACE-1 protein, the position that was most suitable for binding was selected to design a drug with the highest specificity for the BACE-1 protein. This process can be summarized as follows: the ZINCPharmer [36] online service (https://zincpharmer.csb.pitt.edu/pharmer.html) was used to obtain the pharmacophore model for BACE-1, based on its crystallographic structure (PDB ID: 4IVT) and compounds with a specified target. The ZINCPharmer program searched the ZINC database for hit compounds using a pharmacophore model developed from the best binding location of BACE-1 as determined by CavityPlus. A pharmacophore is a 3D arrangement of molecular characteristics within a ligand that are deemed essential for optimal binding interactions with the target protein. The pharmacophore hypotheses were selected according to Lipinski's rule of five to retrieve a diverse set of small molecules from the ZINC database. The radius of the hydrogen bond donor/acceptor feature was 1 Å, while that of the hydrophobic feature was 1.5 Å. Following the selection of pharmacophore features, a small-molecule screening was conducted using ZINCPharmer. The screening results were saved in SDF file format for further analysis.

### 2.2. Ligand–Protein Interactions

The ligand-targeted enzyme dockings were developed to assess the effectiveness of a compound as a BACE-1 inhibitor (PDB 4IVT) compared to Donepezil, which is a typical treatment for AD. ZINCPharmer hit compounds and five modified compounds with improved binding were analyzed, and their 3D structures were created using PubChem (https://pubchem.ncbi.nih.gov//edit3/index.html) (Tables 1 and 2). The structures were optimized using the UFF force field in Avogadro 1.2.0 [37], and the energy of the 3D molecular structures was reduced using Argus Lab [38]. Kollman charges were used to calculate the charge of the polar hydrogens that were included in BACE-1. All of the best ligands for binding to BACE-1 were docked into the enzyme's active site with the use of Argus Lab, AutoDock Vina, and AutoDock.

The docking was considered effective if the binding energy was lower than or equal to that of Donepezil-BACE1. The parameters used to estimate the binding energies for the BACE-1 were a 20 × 24 × 24 box with coordinates *x* = 8.316, *y* = 29.077, *z* = 21.788, and a spacing of 0.403. The exhaustiveness was set to 24 and the grid spacing was 1 Å in AutoDock Vina [39]. AutoDock was used with a 48 × 58 × 58 box, with the same *x*, *y*, and *z* coordinates as the Vina setting, to confirm the binding affinity. Hydrogen bonding and hydrophobic interactions between the chosen compounds and the BACE-1 pocket were visualized using Discovery Studio.

### 2.3. Molecular Dynamic Simulation

MD simulation was used to assess the binding stability of the M4-BACE1 and Donepezil-BACE1 complexes after molecular docking. The PRODRG service was used to create topological data for protein–ligand complexes [40]. In order to conduct this study, we utilized the WebGRO [41] for Macromolecular Simulations program, which solvated the system in a water model, neutralized it, and then added a 0.15 M NaCl salt using the GROMOS96 43a1 force field settings [42]. The steepest descent approach with 5000 steps was utilized, along with constant quantity, volume, temperature (NVT/NPT), and pressure equilibration types, to reduce energy consumption; 1000 ps was chosen as the simulation time, with 1000 frames per simulation, 310 K as the temperature, and 1.0 bar as the pressure [43]. The most important simulation parameters were analyzed, including the root-mean-square fluctuation (RMSF), root-mean-square deviation (RMSD), radius of gyration (Rg), and intermolecular H-bonding (H-bonds).

### 2.4. MM/PB(GB)SA Calculations

The actual binding alignment of the ligands was determined by rescoring docked poses using a web-based tool (accessed on 11 January 2023 at https://cadd.zju.edu.cn/farppi/submit/). Molecular mechanics/Poisson–Boltzmann surface area (MM/PBSA) and molecular generalized Born surface area (MM/GBSA) techniques were used to determine the binding energy of M4-BACE1. These methods included PB1, PB3, PB4, GB1, GB2, GB5, and GB6. Different varieties of Poisson–Boltzmann computations are indicated by the PB and GB notations [44]. In order to determine the binding free energies of the M4-BACE1 and Donepezil-BACE1 complexes, the MM/PB(GB) SA technique was applied.

### 2.5. Retrosynthetic Predictions

When process chemistry is aided by computation, an active pharmaceutical ingredient (API) can be manufactured in a more efficient, less hazardous, and cheaper manner by utilizing fewer processes and cheaper reactants. Retrosynthetic analysis is a common computational tool used for redesigning chemical synthesis. This approach deconstructs the desired chemical into simpler precursor molecules in order to find commercially available starting compounds and synthesis routes. In modern retrosynthesis analysis, artificial intelligence (AI) models are trained on millions of previously analyzed chemical processes. The use of the CAS SciFinder (https://scifinder.cas.org) confirmed the ease with which a fascinating chemical could be synthesized using retrosynthetic analysis [45]. As a result of retrosyntheses and reaction-based enumeration, the newly synthesized chemicals become more readily available using this method.

### 2.6. The Prediction of NMR Chemical Shifts Based on the Structural Compound

Accurate chemical shift predictions for proton NMR were achieved through computational analysis. This involved creating the structures for each synthesis step using the NMR peak prediction program ChemAxon Reactor 22.13.0 [34]. To do this, the compound structures from Tables 1 and 2 were written as a 2D structure, and these structures were then used for proton NMR spectrum prediction.

### 2.7. ADME Studies

The ADME study utilized the SWISS ADME predictor [46], a free web-based tool that assesses the pharmacokinetics and drug-likeness of small molecules. The tool integrated data from multiple programs, including Lipinski, Ghose, Veber, Egan, and Muegge, to provide information on the drug-likeness of the compounds. The study aimed to develop compounds that adhere to the rule of drug-likeness, which involves meeting criteria such as molecular weight less than 500 g/mol, less than 5 hydrogen bond donors, less than 10 hydrogen bond acceptors, and less than 10 rotatable bonds. When evaluating the lipophilicity of druggable compounds, the Lipinski rule sets a maximum limit of 5, which is determined by taking the logarithm of the ratio of the concentration of the compound in two solvents in an uncharged form. An increase in lipophilicity corresponds to a decrease in log P. Water solubility significantly affects a compound's absorption and distribution properties, with poorly soluble compounds being absorbed less efficiently. It is recommended that XLOGP3 falls within the range of −0.7 to 6.0 and that the topology polar surface area (TPSA) is between 20 and 130 Å^2^ [47]. In addition, solubility is expressed as the base-10 logarithm of the solubility in mol/L, referred to as Log S. The optimal Log S distribution for efficient drug uptake and distribution in the body is not higher than 6 [46].

## 3. Results and Discussion

### 3.1. Pharmacophore Modeling

**Figure 1:**
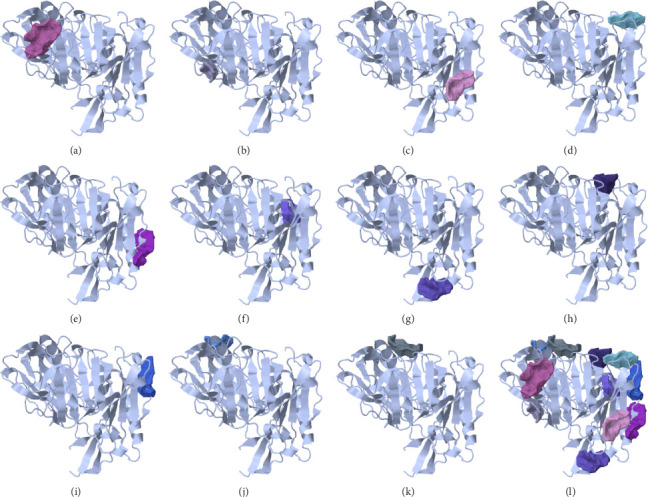
Mapping the covalent-binding site and topography of cavities in the beta-secretase (PDB ID: 4ivt) using cavityplus: a comprehensive analysis of high (a)-to-low (k) prediction max Pkd cavities and all pocket binding sites (l) of BACE1.

**Figure 2:**
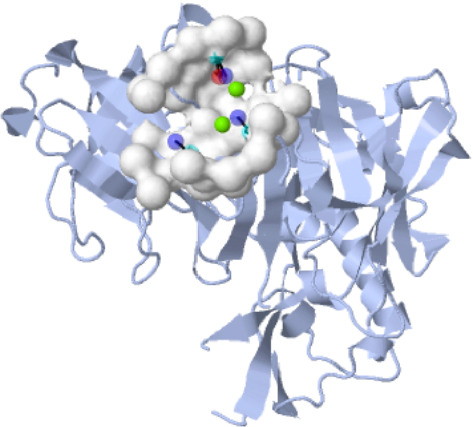
Maximizing ligand binding affinity through identification of high-affinity pharmacophores and pocket binding coordinates: insights from pocket binding site 1.

No research has identified the optimal binding site for BACE-1 inhibitors. In order to determine the most likely binding site and the matching residues, this investigation made use of the Cavity module of the CavityPlus web server. Eleven anticipated cavities were given in Table 3 and Figure 1; the one with the highest drug score and maximum druggability (Figure 1(a)) was selected for further study. Important pharmacophores and their coordinates are shown in Figure 2 and Table 4; they were calculated using the CavPharmer module of the CavityPlus web server.

To guarantee proper absorption and excretion rates, we computationally screened the ZINC database using the pharmacophores, with a molecular weight restriction of 50–500 g/mol [48]. We identified the surface of the receptor as an excluded zone to avoid docking-time clashes with protein side chains. The top two ligand hits were superimposed with the pharmacophore, as illustrated in Table 1, and the ligand with the highest hit score, ZINC35883784, was retrieved and docked to BACE-1. However, the top ligand demonstrated poor bonding when compared to the positive control, prompting adjustments to enhance its inhibition and absorption properties through the gastrointestinal tract and blood–brain barrier, ultimately optimizing its targeted effectiveness. As a result, we developed and improved five new structures, as presented in Table 2.

At present, the primary objective of treating AD is to identify novel inhibitors of BACE1 capable of impeding the progression of the disease through the suppression of toxic amyloid production and neuronal cell loss induced by BACE1 [49]. Thus, inhibiting BACE1 is recognized as a promising approach to developing efficacious neuroprotective drugs designed to interact with the BACE1 pocket and mitigate the deleterious consequences of AD. The in silico approach is a valuable evaluation method for screening a diverse range of target compounds and modified derivatives to identify potential inhibitors of the BACE1 receptor [50]. Molecular docking techniques [51] can expedite the identification of promising BACE1 inhibitors by assessing the interaction binding affinities between the target receptor and a wide array of ligands designed by researchers. This approach offers a significant advantage over conventional screening methods by facilitating rapid identification of potential drug candidates and reducing time and cost expenditures. After analyzing the Zinc database, which encompasses 206,444,075 conformational structures of 21,777,093 compounds, it was determined that only two compounds, namely ZINC35883784 and ZINC19868809, are capable of effectively binding to BACE-1. Table 1 reveals that ZINC35883784 exhibits a more desirable RMSD value of 0.438 than ZINC19868809's RMSD value of 0.472, as its conformational structure is more closely associated with BACE1 [52]. As a result, ZINC35883784, referred to as the “mother compound,” was chosen for further investigation and compared to Donepezil's binding through molecular docking.

### 3.2. Ligand–Protein Interactions

In the process of creating pharmaceuticals for the treatment of AD, molecular docking is essential for selecting viable candidates based on their binding affinity to the BACE1 receptor target. In order to block the BACE1 receptor, docking experiments were undertaken on the mother compound and five modified compounds. The target BACE1 receptor was docked with six ligand molecules, and their binding affinity was ranked. GLY-3, SER-2, PHE-1, VAL0, GLU1, MET2, VAL3, SER86, ILE87, PRO88, HIS89, GLY90, PRO91, ASN92, VAL93, HIS145, VAL146, PRO147, ILE175, ILE176, GLY177, and GLY178 were identified to be the optimal binding sites and are included in Table 3. Based on the most negative number (highest binding energy), the ligand with the strongest binding affinity was determined [53]. The binding affinity of the ligands was compared to that of donepezil using comparative analysis (Table 5). The binding interactions of six compounds (the mother compound and five modified compounds) with the BACE1 receptor ranged from −6.6 to −7.5 kcal/mol.

The binding affinity scores of the hit compounds for suppressing the BACE1 receptor, including the positive control donepezil, are shown in Table 5. Donepezil, with a binding affinity score of −7.7 ± 0.86 and a *p*-value less than 0.05, is the most promising hit chemical for suppressing the BACE1 receptor, according to the data. The modified molecules M1 (−7.4 ± 1.12 kcal/mol), M2 (−7.3 ± 1.39 kcal/mol), M3 (−7.3 ± 1.06 kcal/mol), M4 (−7.5 ± 1.56 kcal/mol), and M5 (−7.3 ± 1.64 kcal/mol) showed high binding affinity. Remarkably, M4 and donepezil displayed equivalent binding affinity scores, with nonsignificant *p*-values (*p*=1), indicating that both drugs had comparable binding capabilities to BACE1.

**Figure 3:**
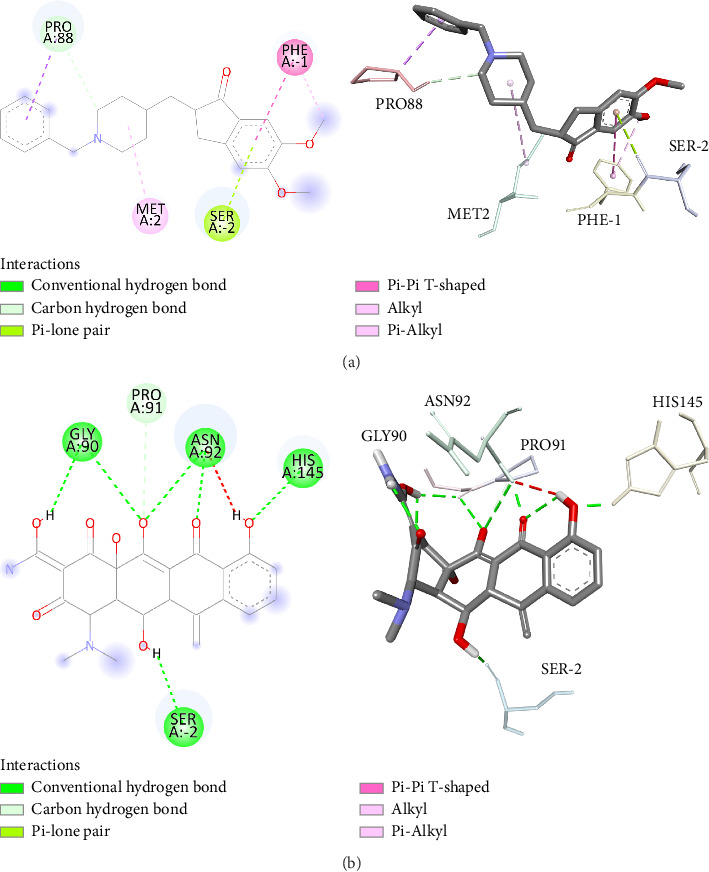
Insights into ligand–receptor binding interactions of (a) donepezil and (b) (1R,3Z,4aS,12R,12aR)-3-[amino (hydroxy)methylidene]-1-(dimethyl amino)-4a,7-dihydroxy-12,12a-dimethyl-11-methylidene-2,4,6-trioxo-1,2,3,4,4a,6,11,11a,12,12a-decahydrotetracen-5-olate with BACE1: a ligand–receptor binding simulation using Discovery Studio Visualizer.

**Figure 4:**
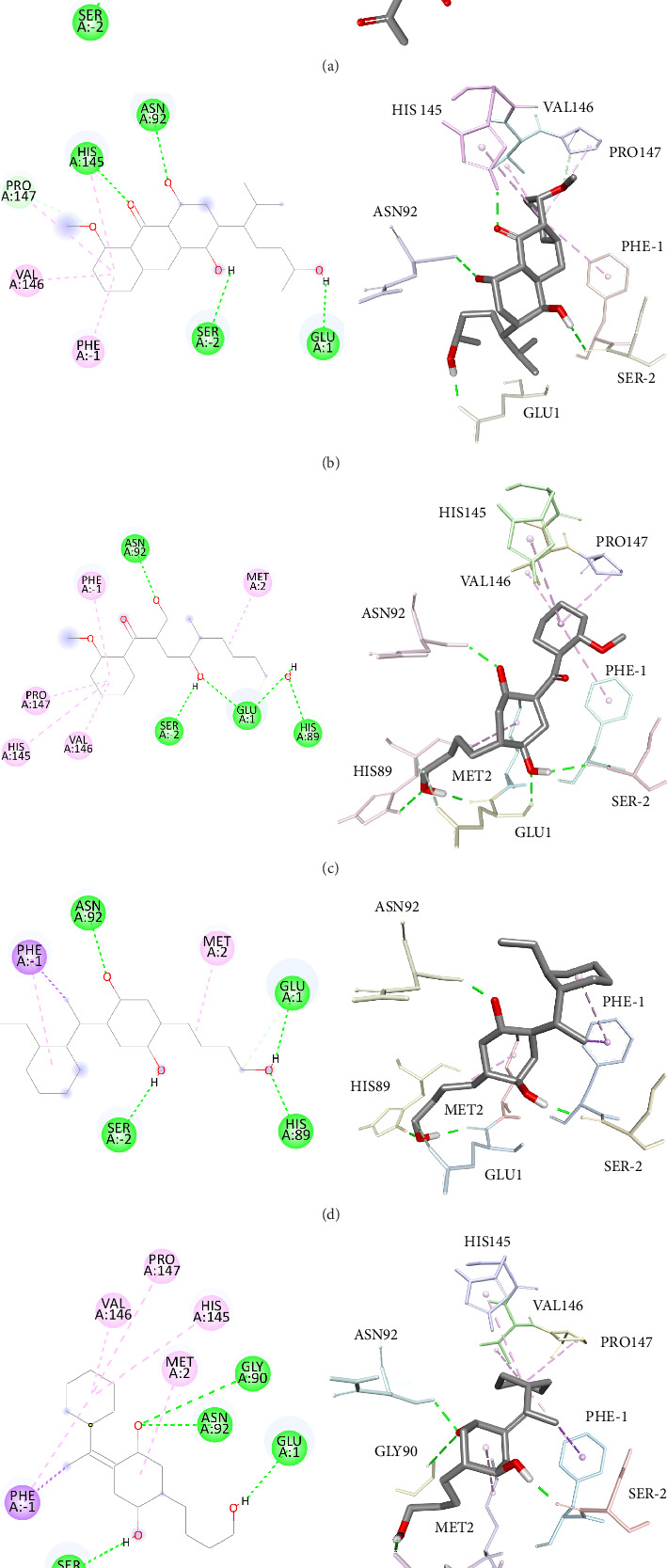
The Discovery Studio Visualizer was utilized to conduct a simulated ligand–receptor interaction analysis for the beta-site amyloid precursor protein cleaving enzyme 1 (BACE1) with five different ligands, namely (a) M1, (b) M2, (c) M3, (d) M4, and (e) M5. The resulting ligand–receptor complex was then visualized in three dimensions (left), with the critical residues being marked in the 2D representation of the ligand–receptor interaction (right).

The present study aimed to identify the amino acid interactions between the BACE1 receptor and the most promising ligands, as presented in Figures 3 and 4. As shown in Table 5, all of the drugs tested exhibited binding activity in the optimal drug-binding site (position 1: Figure 1(a)). In particular, Compound M exhibited binding to six hydrogen bond residues, including SER-2, GLY90, ASN92, and HIS145 amino acid residues, despite the presence of unfavorable donor components in the structure. To enhance the inhibitory efficacy of BACE1, unsuitable structural components were eliminated, resulting in the generation of five novel modified structures illustrated in Figures 4(a), 4(b), 4(c), 4(d), 4(e). Following the implementation of structural modifications, it was discovered that the resultant modified compound structure demonstrated superior binding to BACE1, as indicated in Table 5. It was determined that M4, possessing a binding energy value of −7.5 ± 1.56 kcal/mol comparable to that of Donepezil, represents the most favorable structure for BACE1 inhibition. Of note, as depicted in Figure 4(d), M4 compound structure that is capable of forming hydrogen bonds with BACE1 at four specific positions, namely SER-2, GLU1, HIS89, and ASN92. In contrast, no hydrogen bonding interactions were observed for Donepezil as shown in Figure 3(a). Based on these findings, it is reasonable to conclude that the structurally modified M4 is a viable candidate for inhibiting BACE1. However, there is currently a lack of information regarding the potential neuroprotective effects of the modified compound structure, and further investigation is required in this regard. Moreover, there exists no direct evidence to support the neuroprotection activity of this modified structure. In accordance with the findings presented in Figure 4, it has been ascertained that the cyclohexane-1,4-diol structure represents a crucial configuration for the formation of hydrogen bonds with BACE1, incorporating the critical binding positions of SER-2, GLU1, GLY90, and ASN92. As a result, it is reasonable to deduce that the cyclohexane-1,4-diol structure constitutes a compound framework that holds potential for the development of drugs aimed at conferring neuroprotective effects through the inhibition of BACE1. Nonetheless, it is imperative to conduct further investigations regarding the stability of the binding through the use of molecular dynamics.

### 3.3. Molecular Dynamic Simulation

**Figure 5:**
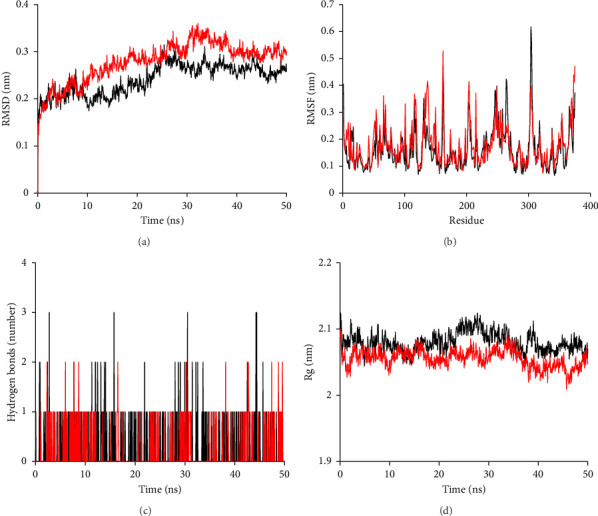
RMSD, RMSF, the total number of hydrogen bonds, and Rg analysis of (red) donepezil and (black) M4 complexes with BACE1 for 50 ns MD simulations. (a) Root-mean-square deviation (RMSD) of Ca atoms. (b) Values of the RMSF for the alpha carbon throughout the entire simulation. (c) Numbers of all H-bonds throughout the simulation. (d) Radius gyration (Rg) throughout the entire simulation.

The investigation of molecular dynamics is a valuable tool for gaining insight into the structure, relationships, and dynamics of biological macromolecules [54]. The results presented in Figure 5(a) indicate that, prior to 25 ns, the RMSD values for both the BACE1:M4 and BACE1:donepezil complexes were unstable and fluctuated around their equilibrium values until the end of the simulation session. Specifically, the mean values for the RMSD values of the BACE1:M4 complex and BACE1:donepezil complex were 0.241 ± 0.033 and 0.278 ± 0.043, respectively, and they fluctuated around these values. The BACE1:M4 complex's surprisingly steady RMSD value throughout the molecular dynamics simulation suggested that the complex had reached equilibrium [55, 56]. The BACE1:donepezil complex, on the other hand, had a greater RMSD value, indicating that its structure was less stable. As shown in Figure 5(b), M4 and donepezil both increased the RMSF value, an indication of residue flexibility [57]. The BACE1:M4 complex had a smaller oscillation pattern throughout the simulation (0.164 ± 0.177 nm) compared to the BACE1:donepezil complex (0.177 ± 0.076 nm), indicating that the BACE1:M4 complex experienced fewer movements.

Figure 5(d) shows the results of an investigation of BACE1's compactness using its Rg factor when in the presence of M4 and donepezil. The oscillation of the Rg factor was found to be 2.055 ± 0.013 nm for the BACE1:donepezil complex and 2.080 ± 0.013 nm for the BACE1:M4 complex. Similar structural alterations occurred throughout the simulation for both the BACE1:M4 complex and the BACE1:donepezil complex, as shown in Figure 5(d). This radius of gyration is consistent with a comparable level of compactness [58] between the BACE1:M4 complex and the BACE1:donepezil complex. As can be observed in Figure 5(c), hydrogen bonds were also studied for both complexes (BACE1:M4 and BACE1:donepezil) since they are an important factor in protein stability [59]. During the course of the simulation, three and two hydrogen bonds were seen in the BACE1:M4 and BACE1:donepezil complexes, respectively. The hydrogen bonding analysis suggests that the BACE1:M4 complex is more stable than the BACE1:donepezil complex.

### 3.4. MM/PB(GB)SA Calculations

To investigate binding structures thoroughly, FarPPI [60] employs the MM/GBSA method to assess the binding free energy of a ligand–protein complex at the level of individual residues. According to the findings of the MM/GBSA calculations, the binding affinity between the ligand and the target protein molecule has increased. Our analysis demonstrates that BACE1 has a greater affinity for binding to M4 than donepezil. FarPPI is able to recognize optimal binding conformations and estimate the appropriate free binding energy scores. Important residues at the binding interface of ligand–protein complexes have been identified by using the per-residue energy decomposition process intrinsic to the MM/GBSA approach.  Table 6 depicts the free binding energy rescoring profiles for M4 and donepezil bound to BACE1 in our investigation, which indicate the strength of the binding relationship. The MM/GBSA approach has proven to be quite effective for rescoring post-docking analytical results. The MM-PB (GB)SA method is effective for predicting binding energies and displaying precise binding structures. The results of the MM-PBSA calculations, as shown in Table 6, suggest that the mean and standard deviation of the binding energy between BACE1 and each ligand are in agreement with the results of previous docking and MD simulations. Importantly, M4 exhibited a more favorable binding behavior to BACE1 than donepezil, as shown by lower binding energies. In terms of MM-PBSA binding energy, our results indicate that M4 is a good option for the development of competitive inhibitors of BACE1, since it demonstrated the most promising results.

### 3.5. Retrosynthetic Analysis and Structural Verification Using NMR

**Figure 6:**
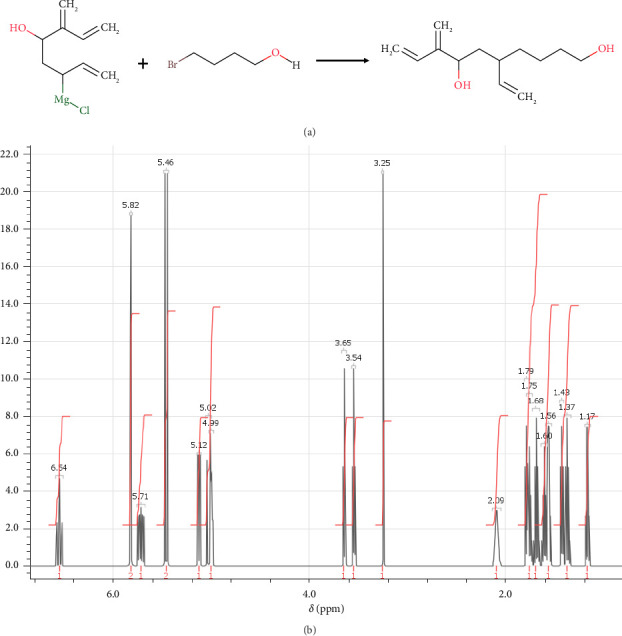
(a) Chemical reactions of (6R)-6-ethenyl-10-hydroxy-3-methylidenedec-1-en-4-olate on a chemical level. (b) ^1^H-NMR spectrum: (ppm) 6.54 (m, 1H-1), 5.82 (q, 2H-2), 5.71 (o, 1H-3), 5.46 (d, 2H-4), 5.12 (q, 1H-5), 5.02 (q, 1H-6), 4.99 (m, 1H-7), 3.65 (t, 1H-8), 3.54 (t, 1H-9), 3.25 (s, 1H-10), 2.09 (m, 2H-11), 1.79 (q, 1H-12), 1.75 (n, 1H-13), 1.68 (p, 1H-14), 1.60 (n, 1H-15), 1.56 (sx, 2H-16), 1.43 (q, 1H-17), 1.37 (p, 1H-18), 1.17 (sx, 1H-19).

The structure of M4 compound was synthesized through a 6-step process as follows. In step 1, (6R)-6-ethenyl-10-hydroxy-3-methylidenedec-1-en-4-olate was synthesized through the reaction previously described by Machara et al. [61]. The synthesis of (6R)-6-ethenyl-10-hydroxy-3-methylidenedec-1-en-4-olate was achieved through a multistep process. In the first step, 6-(chloromagnesio)-3-methylideneocta-1,7-dien-4-olate (1 M solution in THF, 4.2 mL; 4.2 mmol) was added dropwise to a solution of CuCl (0.009 g; 0.09 mmol) and 4-bromo-1-butanol (0.53 g; 1.85 mmol) in tetrahydrofuran (5 mL) to form an intermediate species that subsequently underwent nucleophilic substitution by the bromo substituent of the 4-bromo-1-butanol molecule, resulting in the formation of (6R)-6-ethenyl-10-hydroxy-3-methylidenedec-1-en-4-olate (Figure 6(a)) at room temperature. After 4 h of stirring, the reaction mixture was quenched with aqueous NH_4_Cl and extracted with ethyl acetate to remove the inorganic byproducts and neutralize any residual reactive species. The extraction of the reaction mixture with ethyl acetate allowed for the isolation of the desired product from the reaction mixture. The washing of the organic layer with brine served to remove any remaining inorganic impurities. The concentration of the mixture in vacuo was likely used to remove the solvent and concentrate the product for further purification. Last but not least, (6R)-6-ethenyl-10-hydroxy-3-methylidenedec-1-en-4-olate was isolated in its purest form by separating the residue using column chromatography with cyclohexane/ethyl acetate as the eluent. Overall, this multistep process was successful in synthesizing (6R)-6-ethenyl-10-hydroxy-3-methylidenedec-1-en-4-olate, which can be characterized by NMR spectroscopy, and the spectrum is shown in Figure 6(b) Then, it allowed the formation of (6R)-6-ethenyl-10-hydroxy-3-methylidenedec-1-en-4-olate necessary for step 2 in the synthesis.

**Figure 7:**
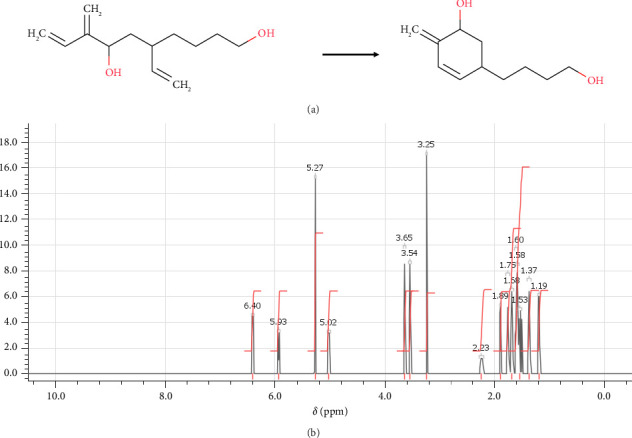
(a) Chemical reactions of (5R)-5-(4-hydroxybutyl)-2-methylidenecyclohex-3-en-1-olate. (b) ^1^H-NMR spectrum: (ppm) 6.40 (q, 1H-1), 5.93 (m, 1H-2), 5.27 (q, 2H-3), 5.02 (m, 1H-4), 3.65 (t, 1H-5), 3.54 (t, 1H-6), 3.25 (s, 1H-7), 2.23 (m, 2H-8), 1.89 (q, 1H-9), 1.75 (n, 1H-10), 1.68 (p, 1H-11), 1.60 (n, 1H-12), 1.58 (sx, 1H-13), 1.53 (q, 1H-14), 1.37 (p, 1H-15), 1.19 (sx, 1H-16).

In step 2, (5R)-5-(4-hydroxybutyl)-2-methylidenecyclohex-3-en-1-olate was synthesized through the reaction previously described by Selander et al. [62]. It involved a multistep process that was carried out to obtain the desired product as shown in Figure 7(a).

In this instance, a ring-closing metathesis reaction was performed on the molecule utilizing Grubbs's second-generation catalyst. The following actions comprised this stage: Under an argon environment, (6R)-6-ethenyl-10-hydroxy-3-methylidenedec-1-en-4-olate was dissolved first. After adding the Grubbs second-generation catalyst, the mixture was agitated for 2 h at 40°C. This was a crucial stage since it provided the way for the production of a cyclic molecule through a ring-closing metathesis reaction. The solvent was then evaporated from the reaction mixture, leaving behind the crude product. Finally, the product was purified by column chromatography, which involved the separation of different components in the mixture based on their chemical properties. Overall, the successful completion of this step was a critical milestone in the synthesis of (6R)-6-ethenyl-10-hydroxy-3-methylidenedec-1-en-4-olate, it can be characterized by NMR spectroscopy, and the spectrum is shown in Figure 7(b) Then, it allowed the formation of the cyclic compound necessary for step 3 in the synthesis.

**Figure 8:**
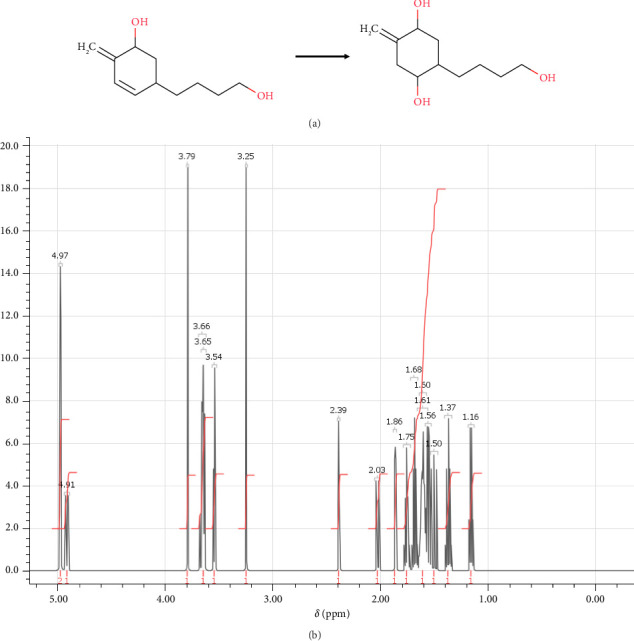
(a) Chemical reactions of (4R,5R)-4-hydroxy-5-(4-hydroxybutyl)-2-methylidenecyclohexan-1-olate. (b) ^1^H-NMR spectrum: (ppm) 4.97 (m, 2H-1), 4.91 (m, 1H-2), 3.79 (s, 1H-3), 3.66 (o, 1H-4), 3.65 (t, 1H-5), 3.54 (t, 1H-6), 3.25 (s, 1H-7), 2.39 (o, 2H-8), 2.03 (o, 2H-9), 1.86 (q, 1H-10), 1.75 (n, 1H-11), 1.68 (p, 1H-12), 1.61 (m, 1H-13), 1.60 (n, 1H-14), 1.56 (sx, 1H-15), 1.50 (q, 2H-16), 1.37 (p, 1H-17), 1.16 (sx, 1H-18).

In step 3, (4R,5R)-4-hydroxy-5-(4-hydroxybutyl)-2-methylidenecyclohexan-1-olate was synthesized through the reaction previously described by Rarig, Scheideman, and Vedejs [63]. It involved a multistep process that was carried out to obtain the desired product as shown in Figure 8(a). The synthesis of (4R,5R)-4-hydroxy-5-(4-hydroxybutyl)-2-methylidenecyclohexan-1-olate can be achieved through a series of reactions. In the first step, the (6R)-6-ethenyl-10-hydroxy-3-methylidenedec-1-en-4-olate is added to a solution of THF at 0°C containing excess THF·BH3 for reducing alkynes to alkenes. This reaction mixture is stirred for 2 h, and then treated with premixed 20% NaOH to destroy any remaining BH3, which can interfere with subsequent steps. The next step involves the addition of 35% H_2_O_2_ dropwise to the mixture to oxidize the borane byproduct to yield the corresponding alcohol. The reaction mixture is then transferred to a separatory funnel containing brine, and the product is extracted with Et2O. MgSO_4_ is used to dry the mixed organic layers. Silica gel chromatography using 15% EtOAc in hexane is used to purify the product after the organic layers have been concentrated under decreased pressure. Overall, this multistep process was successful in synthesizing (4R,5R)-4-hydroxy-5-(4-hydroxybutyl)-2-methylidenecyclohexan-1-olate, which can be characterized by NMR spectroscopy, and the spectrum is shown in Figure 8(b) Then, it allowed the formation of (4R,5R)-4-hydroxy-5-(4-hydroxybutyl)-2-methylidenecyclohexan-1-olate necessary for step 4 in the synthesis.

**Figure 9:**
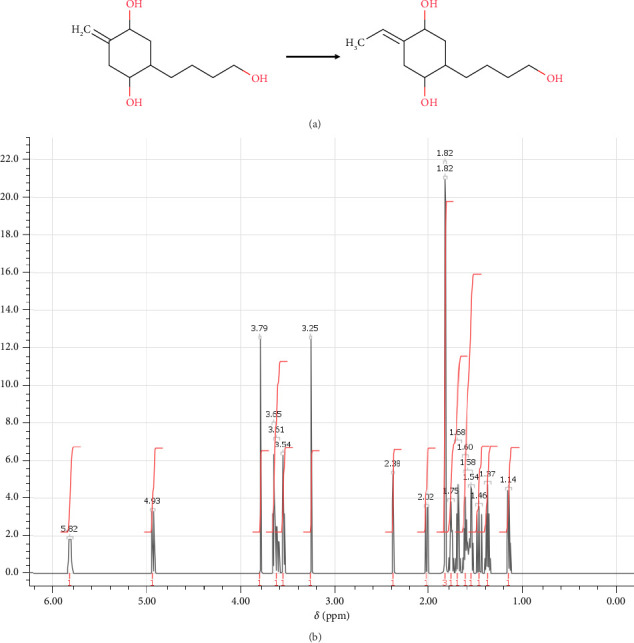
(a) Chemical reactions of (2E,4R,5R)-2-ethylidene-4-hydroxy-5-(4-hydroxybutyl)cyclohexan-1-olate. (b) ^1^H-NMR spectrum: (ppm) 5.82 (m, 1H-1), 4.93 (o, 1H-2), 3.79 (s, 1H-3), 3.65 (t, 1H-4), 3.61 (o, 1H-5), 3.54 (t, 1H-6), 3.25 (s, 2H-7), 2.38 (q, 2H-8), 2.02 (q, 1H-9), 1.82 (d, 3H-10), 1.75 (n, 1H-11), 1.68 (p, 1H-12), 1.60 (n, 1H-13), 1.58 (m, 1H-14), 1.54 (sx, 1H-15), 1.46(q, 1H-16), 1.37 (p, 1H-17), 1.14 (sx, 1H-18).

In step 4, (2E,4R,5R)-2-ethylidene-4-hydroxy-5-(4-hydroxybutyl)cyclohexan-1-olate was synthesized through the reaction previously described by Zhang et al. [64]. In Figure 9(a) The synthesis of (2E,4R,5R)-2-ethylidene-4-hydroxy-5-(4-hydroxybutyl)cyclohexan-1-olate can be successfully done by synthesis and purification of a propylene derivative using Hoveyda–Grubbs second catalyst. The propylene derivative was synthesized by dissolving propylene (54 mg, 0.32 mmol), (4R,5R)-4-hydroxy-5-(4-hydroxybutyl)-2-methylidenecyclohexan-1-olate (412 mg, 3.21 mmol), and Hoveyda–Grubbs second catalyst (40 mg, 0.064 mmol) in degassed DCM (1 mL) in three flasks within an argon-filled glovebox. The solution of propylene was then heated to 40°C. The solution of (4R,5R)-4-hydroxy-5-(4-hydroxybutyl)-2-methylidenecyclohexan-1-olate and Hoveyda–Grubbs second catalyst was added every 5 min into the solution of propylene through respective syringe at the same time in five portions of 0.2 mL each. After the complete addition of (4R,5R)-4-hydroxy-5-(4-hydroxybutyl)-2-methylidenecyclohexan-1-olate and Hoveyda–Grubbs second catalyst, the mixture was stirred for 5 min. After that, low pressure was used to eliminate the solvent. Silica gel column chromatography with PE:EA (1:1) as the eluent was used to refine the initial crude residue. NMR analysis, as shown in Figure 9(b), validated the structure of the produced chemical. Then, it allowed the formation of (2E,4R,5R)-2-ethylidene-4-hydroxy-5-(4-hydroxybutyl)cyclohexan-1-olate necessary for step 5 in the synthesis.

**Figure 10:**
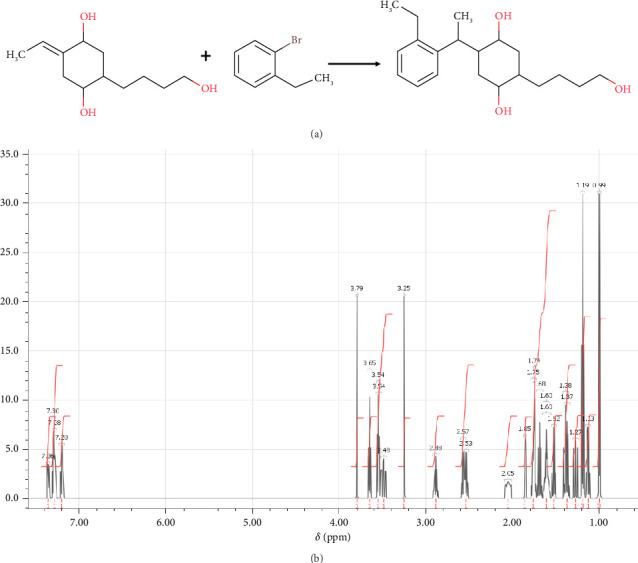
(a) Chemical reactions of (4R,5R)-2-[1-(2-ethylphenyl)ethyl]-4-hydroxy-5-(4-hydroxybutyl) cyclohexan-1-olate. (b) ^1^H-NMR spectrum: (ppm) 7.36 (o, 1H-1), 7.30 (sx, 1H-2), 7.28 (m, 1H-3), 7.20 (sx, 1H-4), 3.79 (s, 1H-5), 3.65 (t, 1H-6), 3.54 (t, 2H-7), 3.48 (o, 1H-8), 3.25 (s, 1H-9), 2.88 (m, 1H-10), 2.57 (o, 1H-11), 2.53 (o, 1H-12), 2.05 (m, 1H-13), 1.85 (q, 1H-14), 1.75 (n, 1H-15), 1.74 (q, 1H-16), 1.68 (p, 1H-17), 1.60 (n, 2H-18), 1.52 (sx, 1H-19), 1.38 (q, 1H-20), 1.37 (p, 1H-21), 1.27 (q, 1H-22), 1.19 (t, 3H-23), 1.13 (sx, 1H-24), 0.99 (d, 3H-25).

In step 5, (4R,5R)-2-[1-(2-ethylphenyl)ethyl]-4-hydroxy-5-(4-hydroxybutyl)cyclohexan-1-olate was synthesized through the reaction previously described by Spino, Gund, and Nadeau [65]. The synthesis of a (4R,5R)-2-[1-(2-ethylphenyl)ethyl]-4-hydroxy-5-(4-hydroxybutyl)cyclohexan-1-olate using various organic synthesis techniques is shown in Figure 10(a). To begin, 100 mg (0.45 mmol) of (2E,4R,5R)-2-ethylidene-4-hydroxy-5-(4-hydroxybutyl)cyclohexan-1-olate was dissolved in 0.5 mL of THF and cooled to 0°C. The reaction mixture was then heated to 80°C for 2 h while a solution of 9-borabicyclononane (1.04 mL, 0.52 mmol) was gently added. To a cooled mixture of 1-bromo-2-ethylbenzene (47 μL, 0.45 mmol), THF (3 mL), and 2 M aqueous solution of potassium carbonate (347 μL, 0.69 mmol), 12 mg of tetrakis(triphenylphosphine)palladium(0) was added. After 18 h of refluxing, water (10 mL) was added to the resulting mixture as it cooled to room temperature. The phases were separated, and 20 mL of diethyl ether was used to extract the aqueous phase three times. Following filtration and drying over anhydrous magnesium sulfate, the organic phases were evaporated at low pressure. The next step was flash chromatography on silica gel purification, with the crude product eluting with a combination of ethyl acetate and hexanes (1:200 to 1:50). This yielded (4R,5R)-2-[1-(2-ethylphenyl)ethyl]-4-hydroxy-5-(4-hydroxybutyl)cyclohexan-1-olate. NMR analysis, as shown in Figure 10(b), validated the structure of the produced chemical. Then, it allowed the formation of (4R,5R)-2-[1-(2-ethylphenyl)ethyl]-4-hydroxy-5-(4-hydroxybutyl)cyclohexan-1-olate necessary for step 6 in the synthesis.

**Figure 11:**
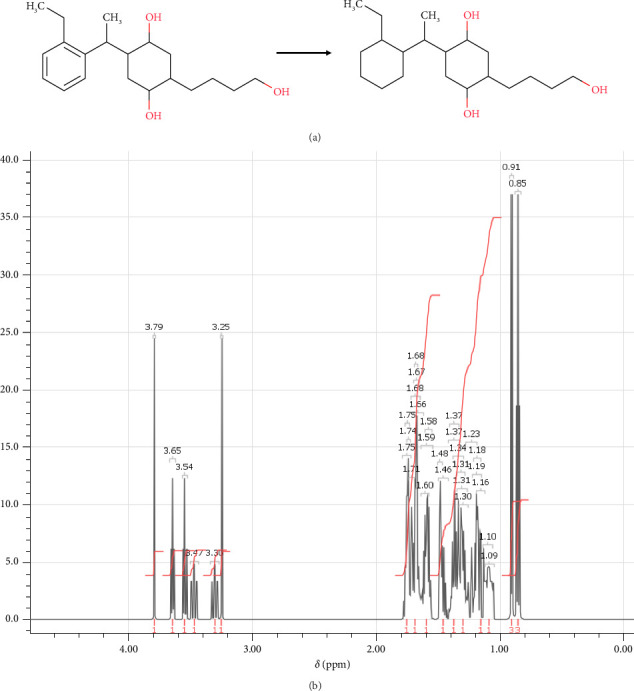
(a) Chemical reactions of (4R,5R)-2-[1-(2-ethylcyclohexyl)ethyl]-4-hydroxy-5-(4-hydroxybutyl) cyclohexan-1-olate. (b) ^1^H-NMR spectrum: (ppm) 3.79 (s, 1H-1), 3.65 (t, 1H-2), 3.54 (t, 1H-3), 3.47 (o, 1H-4), 3.30 (o, 1H-5), 3.25 (s, 1H-6), 1.75 (sx, 2H-7), 1.74 (n, 1H-8), 1.71 (p, 1H-9), 1.68 (q, 1H-10), 1.67 (p, 1H-11), 1.66 (m, 1H-12), 1.60 (n, 1H-13), 1.59 (m, 1H-14), 1.58 (sx, 1H-15), 1.48 (sx, 1H-16), 1.46 (m, 1H-17), 1.37 (p, 1H-18), 1.34 (m, 1H-19), 1.31 (q, 1H-20), 1.30 (o, 1H-21), 1.23 (n, 1H-22), 1.19 (o, 1H-23), 1.18 (sx, 1H-24), 1.16 (q, 1H-25), 1.10 (o, 1H-26), 0.91 (d, 3H-27), 0.85 (t, 3H-28).

In step 6, the last step of the synthesis of the desired compound, (4R,5R)-2-[1-(2-ethylcyclohexyl)ethyl]-4-hydroxy-5-(4-hydroxybutyl)cyclohexan-1-olate (M4) was obtained using a previously described reaction by Snider, Lobera, and Marien [66]. To carry out this reaction, (4R,5R)-2-[1-(2-ethylphenyl)ethyl]-4-hydroxy-5-(4-hydroxybutyl) cyclohexan-1-olate (2.0 mg) was combined with 5% Rh/Al_2_O_3_ catalyst (12 mg), which was used to promote the desired chemical reaction. Rhodium, a transition metal known for its ability to activate hydrogen gas and facilitate hydrogenation reactions, was used in the catalyst. Acetic acid (66 μL) was added to help solubilize the starting material and to assist in the activation of the catalyst, along with EtOH (1.1 mL) to provide a suitable reaction environment. After that, the mixture was mixed for 24 h while pressurized with H_2_ (60 psi). Once the reaction was complete, the mixture was filtered through Celite to get rid of any remaining catalyst. The M4 product (Figure 11(a)) was obtained by concentrating the filtrate. NMR analysis was used to verify the synthesized compound's structure, as indicated in Figure 11(b).

### 3.6. ADME Studies

The analysis suggests that the M and modified structures (M1–M5) have good gastrointestinal absorption, except for M. When compared to Donepezil, which is well absorbed in the gastrointestinal system and can penetrate the blood–brain barrier, three compounds out of the five studied, including M1, M4, and M5, exhibit blood–brain barrier penetration, as shown in Table 7. Additionally, when considering suitable properties such as lipophilicity (based on the XLOGP3 value), the appropriate range is between −0.7 and 6.0, and all studied compounds are within this range except for M. The suitable range for TPSA is between 20 and 130 Å^2^, and all studied compounds fall within this range except for M. The optimal Log S distribution for efficient drug uptake and distribution in the body is not higher than 6, and all studied compounds have a value less than 6, indicating that these compounds can distribute well in the body. The drug-likeness analysis indicates that all modified compounds are suitable for drug synthesis. Overall, based on the analysis, it was found that M1, M4, and M5 are suitable for body absorption and can penetrate the blood–brain barrier, indicating their potential as a treatment for AD.

## 4. Conclusion

The present study utilized receptor-based pharmacophore modeling to identify the optimal binding site and essential pharmacophores for BACE-1 inhibitors. To accomplish this, the Cavity module of the CavityPlus web server was employed to predict the most likely binding site, while the CavPharmer module was utilized to ascertain the essential pharmacophores and their corresponding coordinates. After that, we performed a virtual screen of the ZINC database and docked the best ligand hit, ZINC35883784, onto BACE-1. However, when compared to the positive control, the binding affinity of this ligand was found to be insufficient, necessitating modifications to enhance its inhibitory and absorption capabilities. This led to the creation and improvement of five new compounds. This study has important implications for the development of BACE-1 inhibitors as a therapy for AD. Inhibiting BACE1 is a potential new direction for research into treatments for AD. Potential inhibitors of BACE1 may be screened computationally using in silico molecular docking. Through molecular docking, ZINC35883784 was shown to be a potential inhibitor in this work, and structural adjustments were then conducted to increase its potency. M4, the most effective of the changed structures, inhibited BACE1 activity with a binding affinity that rivaled that of the approved drug Donepezil. However, more research is needed to determine its neuroprotective benefits. In conclusion, molecular docking is a fast and cost-effective method for discovering new treatments for AD. Understanding the structure, relationships, and movements of biological macromolecules requires the use of molecular dynamics simulation. The RMSD value for the BACE1:M4 complex was found to be stable, indicating that the complex had reached equilibrium; in contrast, the RMSD value for the BACE1:donepezil complex was more variable. Furthermore, the BACE1:M4 complex showed less oscillation and fewer fluctuations than the BACE1:donepezil complex, indicating that it suffered fewer structural changes. The BACE1:M4 complex was found to be as compact as the BACE1:donepezil complex, as measured by the Rg factor, and was shown to be more stable than the BACE1:donepezil complex, as measured by the hydrogen bonding study. These results have promising implications for the creation of medications that specifically target BACE1 in the treatment of AD. Insights into the binding affinities of M4 and donepezil to BACE1 were gained via the use of MM/GBSA and MM-PBSA calculations in this investigation. Based on our findings, M4 is a good option for the development of competitive BACE1 inhibitors since it has a higher binding affinity to BACE1 than donepezil. The M4 molecule was synthesized in six stages, with the first being the synthesis of (6R)-6-ethenyl-10-hydroxy-3-methylidenedec-1-en-4-olate. The cyclization in step 2 was crucial for subsequent steps, and the reduction of alkynes to alkenes and purification through silica gel chromatography in step 4 was critical for the process. Finally, M4 was obtained by combining (4R,5R)-2-[1-(2-ethylphenyl)ethyl]-4-hydroxy-5-(4-hydroxybutyl) cyclohexan-1-olate with 5% Rh/Al2O3 catalyst, acetic acid, and EtOH, and shaking the resulting mixture under H2 (60 psi) for 24 h. Throughout the process, NMR spectroscopy was used to confirm the identity and purity of the synthesized compounds. Based on the analysis conducted, it has been demonstrated that M1, M4, and M5 possess favorable physicochemical and pharmacokinetic properties, making them potential candidates for the treatment of AD. These compounds display optimal parameters for gastrointestinal absorption, lipophilicity, and TPSA values, as well as an appropriate Log S distribution, all of which are critical for efficient drug uptake and distribution in the body. Moreover, these compounds exhibit the ability to cross the blood–brain barrier, which is essential for treating AD. The drug-likeness analysis indicates that these compounds are suitable for drug synthesis. Following a thorough evaluation of the pharmacodynamic effects of BACE1 inhibition and the pharmacokinetic properties pertaining to the drug's absorption within the body, the preliminary results indicate that M4 demonstrates promise as a potential therapeutic candidate for the treatment of AD. However, further investigation is imperative to comprehensively ascertain the safety and efficacy profiles of M4. Building upon our computational findings, our next step will be to synthesize M4 following the retrosynthetic pathway we have outlined. This experimental phase will allow us to validate our in silico results and further assess the potential of M4 as a BACE1 inhibitor for AD treatment.

## Figures and Tables

**Table 1:** The basic information about the structure of compounds ZINC35883784 and ZINC19868809.

ZINC ID	ZINC35883784	ZINC19868809
Smiles	CN(C)[C@@H]1[C@H]2[C@@H] ([C@@H]3C(=C)c4cccc(c4C(=O)C3=C([C@@]2(C(=O)/C(=C(/N)\O)/C1=O)O)[O-])O)O	C[C@H]1c2cccc(c2C(=O)C3=C([C@]4([C@H]([C@@H]([C@H]13)O)[C@H](C(=C(C4=O)C(=O)N)[O-])[NH+](C)C)O)[O-])O
IUPAC name	(1R,3Z,4aS,12R,12aR)-3-[amino (hydroxy)methylidene]-1-(dimethyl amino)-4a,7-dihydroxy-12,12a-dimethyl-11-methylidene-2,4,6-trioxo-1,2,3,4,4a,6,11,11a,12,12a-decahydrotetracen-5-olate	(1R,4aR,11R,12R,12aS)-3-carbamoyl-1-(dimethylamino)-7,12-dihydroxy-4a,11-dimethyl-4,6-dioxo-1,4,4a,6,11, 11a,12, 12a-octahydrotetracene-2,5-bis(olate)
Structures	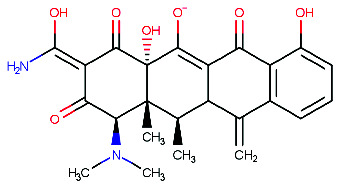	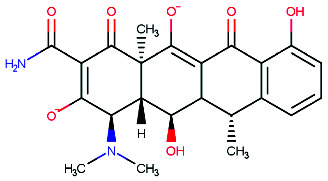
RMSD (Å)	0.438	0.472
Mass (Mw)	442	443

**Table 2:** The basic information about the structure of modified compounds from ZINC35883784.

	Smiles	IUPAC name	Structures
M1	C3(C2[C@@H](C1C(CC(C(=O)C1=C(C2CC(=C(C)C)C3)O)C(C)=O)C)O)C(C)C	(10S)-2-acetyl-9,10-dihydroxy-4-methyl-5-(propan-2-yl)-7-(propan-2-ylidene)-1,2,3,4,4a,5,6, 7,8,8a,10,10a-dodeca hydroanthracen-1-one	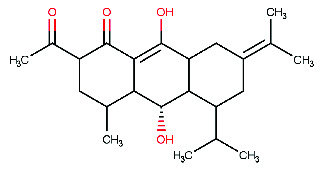

M2	C([H]) ([C@H]3[C@@H]([C@@H]2CC1C(C(CCC1)OC)C(=O)C2C(C3)[O-])O)(CCC(C)O)C(C)C	(3S,4S,4aR)-4-hydroxy-3-(6-hydroxy-2-methyl heptan-3-yl)-8-methoxy-9-oxo-tetradecahydro anthracen-1-olate	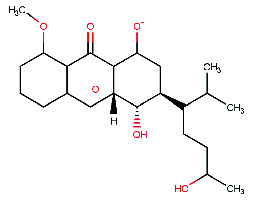

M3	C([H]) ([C@H]2[C@@H](CC(C(C1C(CCCC1)OC)=O)C(C2)[O-])O)CCCO	(4S,5S)-4-hydroxy-5-(4-hydroxybutyl)-2-(2-methoxycyclohexane carbonyl)cyclohexan-1-olate	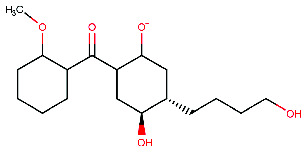

M4	C([C@H]2[C@@H](C([H])C(C(C1CCCCC1CC)C)C(C2)[O-])O)CCCO	(4S,5S)-2-[1-(2-ethyl cyclohexyl)ethyl]-4-hydroxy-5-(4-hydroxy butyl) cyclohexan-1-olate	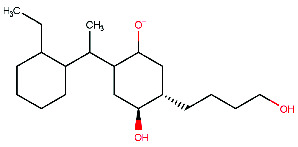

M5	C([C@H]2[C@@H](C([H])\C(=C(/C1CCCCC1)C)C(C2)[O-])O)CCCO	(2E)-2-(1-cyclohexyl ethylidene)-4-hydroxy-5-(4-hydroxybutyl) cyclohexan-1-olate	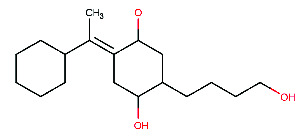

**Table 3:** Predicted cavity analysis, druggability score, and amino acid residues mapping of BACE1.

No	Pred. max pKd	Pred. avg pKd	Drug score	Residues
1	8.80	5.64	−472.00	GLY:−3, SER:−2, PHE:−1, VAL:0, GLU:1, MET:2, VAL:3, SER:86, ILE:87, PRO:88, HIS:89, GLY:90, PRO:91, ASN:92, VAL:93, HIS:145, VAL:146, PRO:147, ILE:175, ILE:176, GLY:177, GLY:178
2	7.84	5.31	−1103.00	VAL:0, GLU:1, MET:2, VAL:3, ASP:4, ASN:5, LEU:6, ARG:7, GLY:8, TYR:15, VAL:16, GLU:17, LEU:27, ASN:28, ARG:50, PRO:88, HIS:89, GLY:90, ASN:114, GLY:172
3	7.48	5.18	−890.00	GLU:207, ILE:208, ASN:209, GLY:210, GLN:211, PRO:281, VAL:282, ILE:283, SER:284, TYR:286, ARG:297, THR:299, ILE:300, LEU:301, GLU:364, PHE:365, ARG:366, THR:367, ALA:368, ALA:369, VAL:370, GLU:371, PHE:374
4	7.38	5.15	−1021.00	TYR:190, THR:191, PRO:192, ILE:193, ARG:194, ILE:202, MET:288, GLY:289, GLU:290, VAL:291, THR:292, ASN:293, GLN:294, THR:376, LEU:377, ASP:378, MET:379, GLU:380, ASP:381
5	7.37	5.15	−1057.00	ILE:208, ASN:209, GLY:210, GLN:211, ASP:212, LEU:213, LYS:214, MET:215, LYS:239, VAL:240, PHE:241, GLU:242, ALA:243, ALA:244, VAL:245, LYS:246, SER:247, ILE:248, LYS:249, ALA:250, ALA:251, SER:252, PRO:281
6	7.28	5.12	−893.00	GLU:219, TYR:220, ASN:221, TYR:222, ASP:223, LYS:224, LEU:236, PRO:237, LYS:238, LYS:239, VAL:240, GLN:326, SER:327, SER:328, THR:329, GLY:330, THR:331, TYR:384
7	7.23	5.10	−1151.00	GLN:271, ALA:272, GLY:273, THR:274, THR:275, PRO:276, TRP:277, ASN:278, ILE:279, GLN:303, PRO:308, ASP:318, TYR:320, VAL:361, HIS:362, ASP:363, GLU:364, PHE:365, ARG:366
8	6.95	5.00	−1042.00	GLU:125, ARG:194, ARG:195, GLU:196, TRP:197, TYR:198, GLU:200, ILE:202, ASN:221, ASP:223, LYS:224, SER:225, THR:329, GLY:330, GLY:383, TYR:384, ASN:385, ILE:386
9	6.83	4.96	−1071.00	ILE:203, VAL:204, ARG:205, VAL:206, GLU:207, ASP:212, LEU:213, LYS:214, MET:215, ASP:216, CYS:217, LYS:218, TYR:220, TYR:286, SER:295, PHE:374, VAL:375, THR:376, LEU:377, ASP:378, MET:379, GLU:380, ASP:381, CYS:382
10	6.52	4.86	−1125.00	ASP:62, LEU:63, ARG:64, LYS:65, GLY:66, LEU:80, GLY:81, THR:82, ALA:97, ASN:98, ARG:128, PRO:129, ASP:130, ASP:131, SER:132, LEU:133, GLU:134, SER:139
11	6.29	4.77	−1263.00	ASN:98, ALA:122, TYR:123, ALA:124, GLU:125, ILE:126, ASP:130, ASP:131, SER:132, LEU:133, GLU:134, PRO:135, PHE:137, ASP:138, SER:139, LEU:140, VAL:141, LYS:142, GLN:143, ASN:148, ARG:195, GLU:196, TRP:197, ASP:346, ARG:347, ALA:348, ARG:349, LYS:350

**Table 4:** Structural determination of cavity 1 in BACE1 and its 3D coordinates for selective ligand binding.

Pharmacophore	*X*	*Y*	*Z*	Radius
H-bond donor center (POK 2.N)	2.50	26.00	24.50	1.00
H-bond root (POK 3.F)	3.35	24.29	26.52	1.50
H-bond acceptor center (POK 2.O)	8.00	34.00	26.00	1.00
H-bond root (POK 3.F)	7.58	36.38	26.02	1.50
Hydrophobic center (POK 2.C)	10.00	32.50	26.50	1.50
Hydrophobic center (POK 2.C)	7.50	28.00	27.00	1.50
H-bond donor center (POK 2.N)	8.50	34.00	26.50	1.00
H-bond root (POK 3.F)	7.58	36.38	26.02	1.50
H-bond donor center (POK 2.N)	8.50	28.50	29.00	1.00
H-bond root (POK 3.F)	9.31	26.60	30.23	1.50

**Table 5:** Calculations of binding energy value, *p*-value significance, number of hydrogen bonds, and interacting residues in inhibitors of BACE1 using computational docking methods (AutoDock, ArgusLab, and AutoDock Vina).

Ligand	Binding energy (kcal/mol)			Avg. binding energy (kcal/mol)	*p*-value	No. of H-bond	Interacting residues
	AutoDock	ArgusLab	Vina				
Donepezil	−7.97	−8.34	−6.7	−7.7 ± 0.86			
Original	−6.81	−6.62	−6.3	−6.6 ± 0.26	0.9135	6	SER-2, GLY90, ASN92, HIS145
M1	−7.04	−8.65	−6.5	−7.4 ± 1.12	0.9999	1	SER-2
M2	−7.51	−8.56	−5.8	−7.3 ± 1.39	0.9997	4	SER-2, GLU1, ASN92, HIS145
M3	−7.32	−8.33	−6.2	−7.3 ± 1.06	0.9995	5	SER-2, GLU1, HIS89, ASN92
M4	−7.7	−9.02	−5.9	−7.5 ± 1.56	1	4	SER-2, GLU1, HIS89, ASN92
M5	−6.48	−9.17	−6.2	−7.3 ± 1.64	0.9997	4	SER-2, GLU1, GLY90, ASN92

**Table 6:** Calculation of binding free energy (in kcal/mol) using MM/PB(GB)SA for the complexes of donepezil-BACE1 and M4-BACE1.

MM/PB(GB)SA	Score (kcal/mol)	
	Donepezil-Bace1	M4-Bace1
PB1	2.55	−11.24
PB3	−8.84	−25.39
PB4	−17.98	−25.39
Avg. PB ± SD	−8.09 ± 10.29	−20.67 ± 8.17
GB1	−25.6	−34.83
GB2	−20.04	−33.2
GB5	−20.93	−34.54
GB6	−13.55	−25.39
Avg. GB ± SD	−20.03 ± 4.96	−31.99 ± 4.46

**Table 7:** Comparison of Swiss ADME pharmacokinetics predictions for six potential BACE1 inhibitor ligands and donepezil.

Properties	Compounds						
	Donepezil	Original	M1	M2	M3	M4	M5
Formula	C_24_H_29_NO_3_	C_22_H_21_N_2_O_8_^−^	C_23_H_34_O_4_^−^	C_23_H_39_O_5_^−^	C_18_H_31_O_5_^−^	C_20_H_37_O_3_^−^	C_18_H_33_O_3_^−^
Molecular weight (g/mol)	379.49	441.41	374.51	395.55	327.44	325.51	297.45
Num. H-bond acceptors	4	9	4	5	5	3	3
Num. H-bond donors	0	5	2	2	2	2	2
TPSA (Å^2^)	38.77	184.45	74.60	89.82	89.82	63.52	63.52
XLOGP3	4.28	0.76	4.03	2.77	1.64	4.71	3.91
Log S (ESOL)	−4.81	−3.13	−4.57	−3.64	−2.44	−4.36	−3.75
GI absorption	High	Low	High	High	High	High	High
BBB permeant	Yes	No	Yes	No	No	Yes	Yes
Lipinski	Yes	Yes	Yes	Yes	Yes	Yes	Yes
Ghose	Yes	No	Yes	Yes	Yes	Yes	Yes
Veber	Yes	No	Yes	Yes	Yes	Yes	Yes
Egan	Yes	No	Yes	Yes	Yes	Yes	Yes
Muegge	Yes	No	Yes	Yes	Yes	Yes	Yes
Bioavailability score	0.55	0.11	0.85	0.56	0.56	0.85	0.85

## Data Availability

All relevant data for this research are provided in the manuscript.
